# The Endothelial Glycocalyx as a Double-Edged Sword in Microvascular Homeostasis and Pathogenesis

**DOI:** 10.3389/fcell.2021.711003

**Published:** 2021-07-14

**Authors:** Nuria Villalba, Sheon Baby, Sarah Y. Yuan

**Affiliations:** ^1^Department of Molecular Pharmacology and Physiology, Morsani College of Medicine, University of South Florida, Tampa, FL, United States; ^2^Department of Surgery, Morsani College of Medicine, University of South Florida, Tampa, FL, United States

**Keywords:** inflammation, microvascular homeostasis, permeability, endothelium, glycocalyx

## Abstract

Expressed on the endothelial cell (EC) surface of blood vessels, the glycocalyx (GCX), a mixture of carbohydrates attached to proteins, regulates the access of cells and molecules in the blood to the endothelium. Besides protecting endothelial barrier integrity, the dynamic microstructure of the GCX confers remarkable functions including mechanotransduction and control of vascular tone. Recently, a novel perspective has emerged supporting the pleiotropic roles of the endothelial GCX (eGCX) in cardiovascular health and disease. Because eGCX degradation occurs in certain pathological states, the circulating levels of eGCX degradation products have been recognized to have diagnostic or prognostic values. Beyond their biomarker roles, certain eGCX fragments serve as pathogenic factors in disease progression. Pharmacological interventions that attenuate eGCX degradation or restore its integrity have been sought. This review provides our current understanding of eGCX structure and function across the microvasculature in different organs. We also discuss disease or injury states, such as infection, sepsis and trauma, where eGCX dysfunction contributes to severe inflammatory vasculopathy.

## Introduction

**FIGURE 1:**
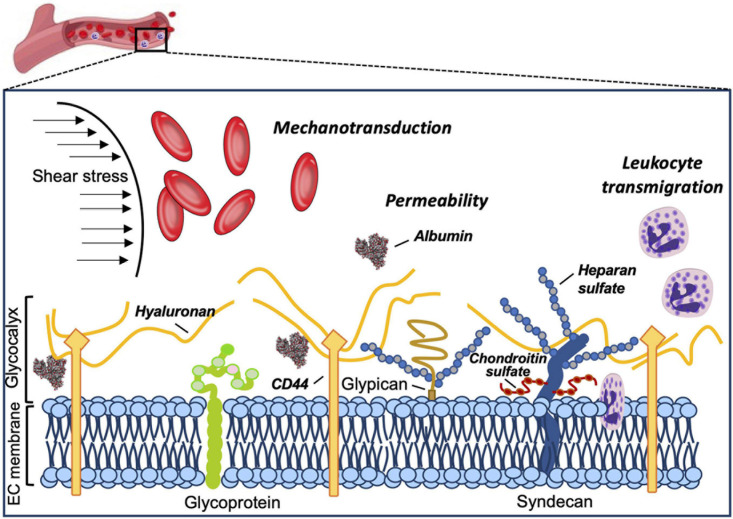
Structure and functions of the eGCX. Schematic representation of the main components and functions of the endothelial glycocalyx. The eGCX is composed of proteoglycans, with long glycosaminoglycan side-chains (GAG-chain) and glycoproteins, with short branched carbohydrate side-chains. The eGCX modulates coagulation, inflammation and mechanotransduction processes.

The vascular endothelial surface is coated by the GCX matrix that confers important functions in circulatory homeostasis (Weinbaum et al., 2007). The endothelial GCX (eGCX), first visualized in the late 1960s after the invention of transmission electron microscope (Luft, 1966), is mainly formed by proteoglycans and glycoproteins, core proteins anchored to the EC membrane that serve as a foundation for the rest of the glycocalyx constituents. Proteoglycans, principally syndecans and glypicans, are decorated by glycosaminoglycan (GAG) chains such as heparan sulfate and chondroitin sulfate (Li et al., 2012). GAGs are characterized by long linear polysaccharides of repeating disaccharide units with a hexosamine and either an uronic acid or a galactose (Esko et al., 2009). The amount of GAG chains, length and molecular modifications by sulfation and/or (de)acetylation provide the eGCX an extensive source of structural rearrangements. Notably, heparan sulfate proteoglycans are the most prominent members expressed on the surface of the endothelial cells, accounting for 50–90% of the total endothelial proteoglycans (Ihrcke et al., 1993). The majority of the interactions between syndecans and extracellular matrix molecules, growth factors and cell adhesion molecules seem to be mediated by their heparan sulfate chains through electrostatic interaction (Bernfield et al., 1992; Stringer and Gallagher, 1997). Unlike other eGCX constituents, hyaluronic acid is a linear, non-sulfated GAG that interacts with the cell surface receptor CD44, a glycoprotein (Aruffo et al., 1990). The glycoproteins are highly branched short carbohydrate chains (2–15 sugar residues) capped with sialic acid or a fucose, which mainly function as either endothelial adhesion molecules or components of the coagulation system (e.g., selectins, immunoglobulins, and integrins) (Figure 1). Further detailed structure and specific components of the eGCX are reviewed elsewhere (Pries and Kuebler, 2006; Tarbell and Pahakis, 2006; Reitsma et al., 2007; Weinbaum et al., 2007; Esko et al., 2009). It is worth noting that the eGCX composition is subject to a highly dynamic regulation and constant replacement or re-arrangement of molecules, ranging from enzymatic degradation (“shedding”) to *de novo* biosynthesis of new molecules and to recruitment of circulating molecules from the blood.

In the following sections, we will focus our discussion on the eGCX as an active component of the EC barrier, its functions, and structural variations within the vascular tree and across organs. Furthermore, we will also summarize the new findings from eGCX research with respect to how eGCX degradation leads to certain vascular pathologies.

### The eGCX: An Active Layer Without a Passive Role

The eGCX matrix is an integral component of the vascular wall. Apart from being a physical barrier, the eGCX also plays an effective role in modulating vascular homeostasis. Historically, the eGCX was considered to function as an additional physical barrier between the vessel lumen and the EC membrane (Curry and Adamson, 2012); however, solid experimental evidence has shown an important physiological role for the eGCX in performing a variety of microvascular functions such as regulating vascular permeability, mechanotransduction and leukocyte transmigration (Ihrcke et al., 1993; Davies, 1995; Baldwin and Thurston, 2001; Constantinescu et al., 2003; Curry, 2005; Tarbell and Ebong, 2008; Lopez-Quintero et al., 2009; Lennon and Singleton, 2011; Curry and Adamson, 2012).

The eGCX is one of the major determinants in maintaining endothelial barrier function by acting as an additional molecular filter for the endothelium. The eGCX modulates vascular permeability and hydraulic conductivity by limiting the flux of water and macromolecules (Curry and Michel, 1980; Adamson, 1990; Curry, 2005; Lennon and Singleton, 2011; Curry and Adamson, 2012). It also acts as a vascular barrier through modulation of molecular binding to the EC surface due to the high density of anionic charges on its GAGs side chains. The net negative charge of the eGCX carried by sulfate residues along the GAG chains favors the docking (adsorption) of positively charged molecules (Schnitzer, 1988; Lieleg et al., 2009). Thus, the eGCX regulates vascular permeability by restricting circulating molecules from strongly attaching to the endothelium based on their net charge. Importantly, the molecular size (70–kDa cutoff) is also relevant in determining the penetration of molecules into the eGCX layer, as much as chemical binding (Henry and Duling, 1999; Vink and Duling, 2000; Curry and Adamson, 2012).

Previous studies using perfusion models or intravital microscopy techniques found that eGCX damage by heparinase causes microvascular leakage (Rehm et al., 2004; Jacob et al., 2006). Similar results were found using genetic knock down of a specific eGCX component (Voyvodic et al., 2014). In this regard, increased hydraulic conductivity (*Lp*) of microvessels after removal of the eGCX or plasma proteins has also been shown (Huxley and Curry, 1985; Adamson and Clough, 1992; Weinbaum et al., 2007).

The eGCX plays a pivotal role in mechanotransduction together with other sensors in the endothelium, including G–protein–coupled receptors (Zou et al., 2004; Mederos y Schnitzler et al., 2008), Piezo and transient receptor potential (TRP) channels (Martinac, 2004; Coste et al., 2010; Dragovich et al., 2016), caveolar structures (Rizzo et al., 1998), and integrins and focal adhesions (Ringer et al., 2017). Blood flow exerts mechanical tangential forces to the endothelial surface such as shear stress, which is sensed by the eGCX and triggers the production of nitric oxide (NO), an important modulator of vascular tone (Davies, 1995; Dimmeler et al., 1999; Tarbell and Ebong, 2008; Fu and Tarbell, 2013; Zeng et al., 2018). The ability of the eGCX to reorganize the actin cytoskeleton under shear forces has been demonstrated in studies using EC monolayers as well as *in vivo* approaches. The eGCX core protein syndecan-1 interacts with cytoskeletal proteins through a highly conserved tyrosine residue in the syndecan family (Carey et al., 1996). Also, syndecan-4 acts synergistically with integrins to assemble and rearrange actin stress fibers to orchestrate cell adhesion and focal contact formation (Echtermeyer et al., 1999; Bass et al., 2007; Multhaupt et al., 2009). Interestingly, while syndecans are the main effector in cell adhesion or shape changes via their interaction with the cytoskeleton, glypicans mediate flow–induced endothelial NO synthase (eNOS) activation, based on their location at the endothelial membrane microdomains where caveolae reside (Ebong et al., 2014; Zeng and Liu, 2016; Bartosch et al., 2017). Prior studies with cultured ECs have shown that breakdown of heparan sulfate alters shear stress and impairs NO production (Florian et al., 2003); similar responses were also observed *in vivo* on canine femoral and rabbit mesenteric arteries, where infusion of hyaluronidase (to degrade hyaluronic acid GAGs) or neuraminidase (to remove sialic acid residues), respectively, reduced flow–dependent vasodilation, which is mediated by NO release (as in the majority of vascular beds) (Pohl et al., 1991; Mochizuki et al., 2003).

Additionally, the eGCX also controls the interaction between the endothelium and circulating cells by preventing the latter from approaching the endothelium under basal conditions. Upon inflammatory stimulation, the glycans are shed from the EC surface allowing slow rolling and adhesion of leukocytes (Constantinescu et al., 2003; Lipowsky et al., 2011). Similarly, breakdown of the eGCX increases platelet–vessel wall interactions, further demonstrating an anti-coagulant effect by the eGCX layer (Vink et al., 2000).

### The Endothelium Is Heterogenous, So Is the eGCX

The morphology of the microvascular endothelium and associated gene expression vary across different vascular beds in different tissues, therefore showing a remarkable heterogeneity (Aird, 2007; Jambusaria et al., 2020). Likewise, different GAG chain arrangements and eGCX compositions result in great biochemical or structural variations, further contributing to the eGCX heterogeneity. With reference to the thickness and microstructure of the eGCX, it is now well established that both vary across different species, vascular beds, organs and shear stress rates.

The estimation of the eGCX thickness extends from 0.2 to 0.5 μm in capillaries (van den Berg et al., 2003) and venules (Yoon et al., 2017), to 2–3 μm in small arteries (van Haaren et al., 2003; Yen et al., 2015), and 4.5 μm in conductance arteries (Megens et al., 2007). These studies used different methods of eGCX visualization and measurements, including alcian blue staining for transmission electron microscopy, dye–exclusion of different sized tracers, and fluorescently labeled lectins for microscopic imaging (Roth, 1983; Vink and Duling, 2000; van den Berg et al., 2003). Still, there is a large discrepancy when it comes to reporting eGCX thickness, making experimental observations particularly difficult to be reconciled. The reason for this variability, which might not be entirely attributed to differences in the microstructure and composition of the eGCX, might rather be due to a poor preservation of such a fragile structure during fixation and tissue handling (de Mesy Bentley, 2011; Ebong et al., 2011). Comparatively, direct *in vivo* measurements using bright-field microscopy also embody challenges. The close optical refractive index of the eGCX to the surrounding blood makes it very difficult to visualize the eGCX limits, also contributing to bias in the results. *In vitro*, ECs in culture exhibit slightly different eGCX in comparison to the complex structure found in *in vivo* vessels (Potter and Damiano, 2008; Potter et al., 2009). Recently, super resolution fluorescence microscopy (STORM) has been applied to identify the spatio-chemical organization of the eGCX *in vitro* (Fan et al., 2019). Also, glycomic analysis by liquid chromatography coupled to mass spectrometry has emerged as a novel method providing a more detailed and comprehensive characterization of eGCX in cells and tissues (Li et al., 2019, 2020; Riley et al., 2020).

A close view of the eGCX using both scanning and transmission electron microscopy has revealed different eGCX thickness among continuous, fenestrated and sinusoidal capillaries in the heart, kidney, and liver, respectively (Okada et al., 2017). The eGCX layer in both continuous and fenestrated capillaries is thicker than in the sinusoids. In the heart, the eGCX covers the entire luminal endothelial surface. In the kidney, the eGCX appears to occlude the endothelial pores of the fenestrated capillaries. In the hepatic sinusoids, however, the eGCX covers both the luminal side and opposite side facing the perisinusoidal space (Okada et al., 2017).

In organs like the brain and heart, where the capillary endothelium is categorized as continuous (non-fenestrated), the endothelial eGCX appears to be denser compared to that in the lung, whose capillaries are also covered by continuous endothelium (Ando et al., 2018). These differences might be explained by the mechanotransduction properties of the eGCX in sensing fluid shear stress, which alters GAGs synthesis (Arisaka et al., 1995; Gouverneur et al., 2006; Zeng and Tarbell, 2014). Since the pulmonary circulation is a low fluid shear stress system (because of its low resistance), a lower rate of GAGs synthesis renders a thinner eGCX on the pulmonary capillaries compared to other organs like the heart or the kidney. However, experimental evidence shows discrepancies in eGCX depth between pulmonary eGC (>1.5 micrometers) exceeding that of systemic vessels such as the eGCX in cremaster muscle capillaries (Schmidt et al., 2012; Han et al., 2016). The same principle can be applied to the macro vs. microvascular network, where arteries receiving higher shear stress exhibit greater eGCX depths compared to venules and capillaries with lower shear stress (Lipowsky et al., 1978, 1980; van den Berg et al., 2003). In light of recent discoveries, differences in capillary EC structure and shear stress might not be sufficient to explain eGCX heterogeneity. Gene expression profiling and single–cell RNA-sequencing might yield a more comprehensive picture of the distinct EC subsets and associated eGCX structures (Jambusaria et al., 2020; Gao and Galis, 2021).

### Severe Inflammation as a Cause of eGCX Dysfunction

**FIGURE 2:**
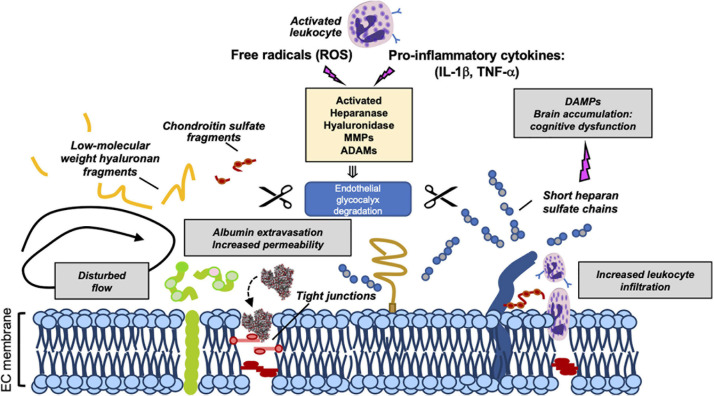
Mechanisms of eGCX degradation and pathogenic consequences of released GCX fragments. Representation of enzymatic degradation of GCX components. The structure of the eGC is the result of a balance between the enzymatic degradation and *de novo* biosynthesis of new molecules and adsorption of circulating components from blood. Several enzymes mediate this degradation. Heparinase, hyaluronidase, MMPs and ADAMs are activated by pro-inflammatory cytokines and ROS promoting the damage and shedding of one or more of its components. This degradation releases eGCX components (such as short heparan sulfate chains, low-molecular weight hyaluronan fragments, and chondroitin sulfate fragments) into the circulation. As a result of its degradation, the eGC becomes thinner allowing the extravasation of albumin, leukocyte adhesion and dysregulated vasodilation. Once in circulation, eGCX components such as heparan sulfate fragments can act as DAMPs leading to cognitive impairment (Hippensteel et al., 2019a). Gray box areas summarize major pathophysiologic features of eGCX degradation. DAMPs, danger-associated molecular patterns; MMP, metalloproteinase; IL-1β, interleukin-1β; TNF-α, tumor necrosis factor-α. Scissors symbol means “degradation”.

Recently, the eGCX integrity has emerged as an important determinant of cardiovascular health and disease. Given the fundamental role of the eGCX in maintaining vascular homeostasis, one would predict that when components of the eGCX are lost or degraded, the endothelial function could be impaired, which has indeed been demonstrated. eGCX degradation is triggered by inflammatory mechanisms through the activation of specific enzymes such as metalloproteinases, heparanase, and hyaluronidase. These enzymes are activated by reactive oxygen species (ROS) and pro-inflammatory cytokines such as tumor necrosis factor alpha (TNF-α) and interleukin-1 beta (IL-1β) (Figure 2) (Chappell et al., 2008; Schmidt et al., 2012; Lipowsky and Lescanic, 2013; Manon-Jensen et al., 2013; Becker et al., 2015).

The lack of an intact eGCX has been observed in several pathological conditions, the best characterized being sepsis. In the broad scheme of sepsis, systemic inflammatory injury of the eGCX leads to capillary leak, adverse immune response, and impaired vasodilation. Following septic challenge, enzymes such as ADAM15 (a disintegrin and metalloproteinase 15) and heparanase can shed glycoproteins (CD44) and heparan sulfate, respectively, leading to eGCX disruption (Schmidt et al., 2012; Yang et al., 2018). As a result of eGCX damage, the eGCX layer becomes thinner and more sparse while its degradation products are released into the bloodstream, a phenomenon that has been observed in animal models of sepsis as well as in human patients with sepsis, trauma or shock (Nelson et al., 2008; Haywood-Watson et al., 2011; Sallisalmi et al., 2012; Luker et al., 2018; Uchimido et al., 2019).

Similar to sepsis, sterile inflammation following trauma or tissue injury also causes shedding of proteoglycans, hyaluronan and heparan sulfate chains. The eGCX fragments function as Danger-Associated Molecules Patterns (DAMPs) that activate toll–like receptor or/and RAGE receptor-dependent pathways (Johnson et al., 2002) RAGE (Xu et al., 2011, 2013). High levels of circulating eGCX elements, which propagate sterile inflammation and drive trauma induced coagulopathy (TIC), are highly correlated with the severity of injury and clinical outcomes (Johansson et al., 2011a, b).

Oxidative stress also plays an important role in eGCX degradation during inflammation. The eGCX along with vascular ECs are vulnerable to circulating ROS produced during oxidative stress. *In vitro* exposure of ROS (superoxide and hydroxyl radicals) to the eGCX promotes fragmentation of GAGs and loss of some of its components. Previous studies have demonstrated that hyaluronan and chondroitin sulfate are the most susceptible to depolymerization and chemical modifications by ROS (Halliwell, 1978; Greenwald and Moy, 1980; Bartold et al., 1984; Moseley et al., 1995, 1997; Lipowsky and Lescanic, 2013; Singh et al., 2013). Intact eGCX has the capability to quench free radicals by having binding sites for anti-oxidant enzymes like xanthine oxidoreductase (Adachi et al., 1993) and endothelial superoxide dismutase (eSOD) (Becker et al., 1994).

Viral infections, such as those caused by dengue, hanta and the novel severe acute respiratory syndrome (SARS)-CoV-2 (COVID-19), are also accompanied by eGCX disruption. In the case of the dengue virus, in particular, the secreted dengue virus (DENV) non-structural protein 1 (NS1) disrupts the eGCX on human pulmonary capillaries by increasing the expression of sialidases, heparanase and metalloproteinases. All these events cause systemic microvascular leakage leading to hypovolemic shock and potentially fatal complications in severe dengue infections (Luplertlop and Misse, 2008; Puerta-Guardo et al., 2016; Glasner et al., 2017; Suwarto et al., 2017; Tang et al., 2017; Chen et al., 2018; Wang et al., 2019). Hantavirus infection is also associated with endothelial dysfunction and elevated circulating levels of syndecan-1, allowing a clinical association of disease severity with eGCX damage (Marsac et al., 2011; Connolly-Andersen et al., 2014). In contrast, other viruses do not seem to cause eGCX shedding, but they exploit eGCX components on the host cell surface as a binding site to infect target cells. For example, Influenza A uses sialic acid as a receptor (Weis et al., 1988; Matrosovich et al., 1993; Suzuki, 2003; Russell et al., 2008) while HIV lentivirus (Saphire et al., 2001; Bobardt et al., 2003; Gallay, 2004) and SARS-CoV-2 (Clausen et al., 2020) interact with heparan sulfate. Also, several recent studies have emphasized the implications of eGCX damage and endothelial dysfunction in the pathogenesis of COVID-19 (Jung et al., 2020; Kaur et al., 2020; Libby and Luscher, 2020; Teuwen et al., 2020; Yamaoka-Tojo, 2020).

Previous research on fluid resuscitation for critical illness management has shown mixed results, some show attenuating eGCX degradation while others show inducing eGCX disruption (Hippensteel et al., 2019b). However, there is consensus that colloids (e.g., albumin), or fresh frozen plasma, reduce eGCX damage following sepsis, hemorrhagic shock and traumatic brain injury (Zehtabchi and Nishijima, 2009; Haywood-Watson et al., 2011; Kozar et al., 2011; Peng et al., 2013; Mica et al., 2016; Nikolian et al., 2018).

### Endothelial GCX in Blood–Brain Barrier (BBB) Injury

The diagnostic utility of eGCX degradation products as a biomarker of disease is supported by the correlation between circulating eGCX fragments and clinical outcomes [reviewed by Uchimido et al. (2019)]. Compared to the cardiac and pulmonary capillaries, cerebral capillaries have a thicker eGCX layer which is better preserved following lipopolysaccharide (LPS) administration (Ando et al., 2018). Additionally, the eGCX joins astrocyte endfeet and basement membrane in reinforcing BBB properties as a part of a newly defined “tripartite” BBB layered structure (Kutuzov et al., 2018). During sepsis, heparan sulfate fragments released from the injured eGCX can circulate in the bloodstream for days and penetrate into the hippocampal area, interfering with long-term potentiation (LTP) and contributing to sepsis–associated encephalopathy (SAE), a common neurological complication of sepsis in the absence of direct brain infection (Hippensteel et al., 2019a). Circulating eGCX fragments predicted cognitive impairment in septic patients, however, whether they have potential diagnostic utility as biomarkers to predict cognitive dysfunction in sepsis survivors, still remains to be confirmed.

## Conclusion

The eGCX, a complex and fragile structure that protects endothelial barrier integrity, plays a crucial role in maintaining microcirculatory homeostasis and blood-tissue exchange. Disruption of eGCX is a consequence as well as cause of microvascular injury, as eGCX degradation products act as pathogenic factors capable of inducing endothelial hyperpermeability and microvascular leakage during inflammation. Further studies are required to understand eGCX structure and function in order to maximize its protective contribution to endothelial cell stability while minimizing its pathological role in vascular disease and injury.

## Author Contributions

NV performed literature search, drafted the manuscript, and prepared the figures. SB and SY participated in manuscript editing. SY initiated, directed, and sponsored the work throughout all levels of development. All authors approved the final version for publication.

## Conflict of Interest

The authors declare that the research was conducted in the absence of any commercial or financial relationships that could be construed as a potential conflict of interest.

## Abbreviations

BBB: blood–brain barrier
DAMP: danger-associated molecular pattern
EC: endothelial cell
eNOS: endothelial nitric oxide synthase
eSOD: endothelial superoxide dismutase
GAG: glycosaminoglycan
eGCX: endothelial glycocalyx
LPS: lipopolysaccharide
NO: nitric oxide
ROS: reactive oxygen species
SAE: sepsis–associated encephalopathy
TRPs: transient receptor potential channels.

## References

[B1] Adachi T. Fukushima T. Usami Y. Hirano K. (1993). Binding of human xanthine oxidase to sulphated glycosaminoglycans on the endothelial-cell surface. *Biochem. J.* 289 523–527. 10.1042/bj2890523 8424793 PMC1132198

[B2] Adamson R. H. (1990). Permeability of frog mesenteric capillaries after partial pronase digestion of the endothelial glycocalyx. *J. Physiol.* 428 1–13. 10.1113/jphysiol.1990.sp018197 2231409 PMC1181632

[B3] Adamson R. H. Clough G. (1992). Plasma proteins modify the endothelial cell glycocalyx of frog mesenteric microvessels. *J. Physiol.* 445 473–486. 10.1113/jphysiol.1992.sp018934 1501143 PMC1179992

[B4] Aird W. C. (2007). Phenotypic heterogeneity of the endothelium: i. structure, function, and mechanisms. *Circ. Res.* 100 158–173. 10.1161/01.RES.0000255691.76142.4a 17272818

[B5] Ando Y. Okada H. Takemura G. Suzuki K. Takada C. Tomita H. (2018). Brain-specific ultrastructure of capillary endothelial Glycocalyx and its possible contribution for blood brain barrier. *Sci. Rep.* 8:17523. 10.1038/s41598-018-35976-2 30504908 PMC6269538

[B6] Arisaka T. Mitsumata M. Kawasumi M. Tohjima T. Hirose S. Yoshida Y. (1995). Effects of shear stress on glycosaminoglycan synthesis in vascular endothelial cells. *Ann. N. Y. Acad. Sci.* 748 543–554. 10.1111/j.1749-6632.1994.tb17359.x 7695202

[B7] Aruffo A. Stamenkovic I. Melnick M. Underhill C. B. Seed B. (1990). CD44 is the principal cell surface receptor for hyaluronate. *Cell* 61 1303–1313. 10.1016/0092-8674(90)90694-A 1694723

[B8] Baldwin A. L. Thurston G. (2001). Mechanics of endothelial cell architecture and vascular permeability. *Crit. Rev. Biomed. Eng.* 29 247–278. 10.1615/CritRevBiomedEng.v29.i2.20 11417757

[B9] Bartold P. M. Wiebkin O. W. Thonard J. C. (1984). The effect of oxygen-derived free radicals on gingival proteoglycans and hyaluronic acid. *J. Periodontal. Res.* 19 390–400. 10.1111/j.1600-0765.1984.tb01012.x 6205132

[B10] Bartosch A. M. W. Mathews R. Tarbell J. M. (2017). Endothelial glycocalyx-mediated nitric oxide production in response to selective AFM pulling. *Biophys. J.* 113 101–108. 10.1016/j.bpj.2017.05.033 28700908 PMC5510764

[B11] Bass M. D. Morgan M. R. Humphries M. J. (2007). Integrins and syndecan-4 make distinct, but critical, contributions to adhesion contact formation. *Soft Matter.* 3 372–376. 10.1039/b614610d 19458789 PMC1828213

[B12] Becker B. F. Jacob M. Leipert S. Salmon A. H. Chappell D. (2015). Degradation of the endothelial glycocalyx in clinical settings: searching for the sheddases. *Br. J. Clin. Pharmacol.* 80 389–402. 10.1111/bcp.12629 25778676 PMC4574825

[B13] Becker M. Menger M. D. Lehr H. A. (1994). Heparin-released superoxide dismutase inhibits postischemic leukocyte adhesion to venular endothelium. *Am. J. Physiol.* 267 H925–H930. 10.1152/ajpheart.1994.267.3.H925 8092297

[B14] Bernfield M. Kokenyesi R. Kato M. Hinkes M. T. Spring J. Gallo R. L. (1992). Biology of the syndecans: a family of transmembrane heparan sulfate proteoglycans. *Annu. Rev. Cell Biol.* 8 365–393. 10.1146/annurev.cb.08.110192.002053 1335744

[B15] Bobardt M. D. Saphire A. C. Hung H. C. Yu X. Van Der Schueren B. Zhang Z. (2003). Syndecan captures, protects, and transmits HIV to T lymphocytes. *Immunity* 18 27–39. 10.1016/S1074-7613(02)00504-6 12530973

[B16] Carey D. J. Bendt K. M. Stahl R. C. (1996). The cytoplasmic domain of syndecan-1 is required for cytoskeleton association but not detergent insolubility. identification of essential cytoplasmic domain residues. *J. Biol. Chem.* 271 15253–15260. 10.1074/jbc.271.25.15253 8662979

[B17] Chappell D. Jacob M. Rehm M. Stoeckelhuber M. Welsch U. Conzen P. (2008). Heparinase selectively sheds heparan sulphate from the endothelial glycocalyx. *Biol. Chem.* 389 79–82. 10.1515/BC.2008.005 18095872

[B18] Chen H. R. Chao C. H. Liu C. C. Ho T. S. Tsai H. P. Perng G. C. (2018). Macrophage migration inhibitory factor is critical for dengue NS1-induced endothelial glycocalyx degradation and hyperpermeability. *PLoS Pathog.* 14:e1007033. 10.1371/journal.ppat.1007033 29702687 PMC6044858

[B19] Clausen T. M. Sandoval D. R. Spliid C. B. Pihl J. Perrett H. R. Painter C. D. (2020). SARS-CoV-2 infection depends on cellular heparan sulfate and ACE2. *Cell* 183 1043–1057 e1015. 32970989 10.1016/j.cell.2020.09.033 PMC7489987

[B20] Connolly-Andersen A. M. Thunberg T. Ahlm C. (2014). Endothelial activation and repair during hantavirus infection: association with disease outcome. *Open Forum Infect. Dis.* 1:ofu027. 10.1093/ofid/ofu027 25734100 PMC4324194

[B21] Constantinescu A. A. Vink H. Spaan J. A. (2003). Endothelial cell glycocalyx modulates immobilization of leukocytes at the endothelial surface. *Arterioscler. Thromb. Vasc. Biol.* 23 1541–1547. 10.1161/01.ATV.0000085630.24353.3D 12855481

[B22] Coste B. Mathur J. Schmidt M. Earley T. J. Ranade S. Petrus M. J. (2010). Piezo1 and Piezo2 are essential components of distinct mechanically activated cation channels. *Science* 330 55–60. 10.1126/science.1193270 20813920 PMC3062430

[B23] Curry F. E. Adamson R. H. (2012). Endothelial glycocalyx: permeability barrier and mechanosensor. *Ann. Biomed. Eng.* 40 828–839. 10.1007/s10439-011-0429-8 22009311 PMC5042904

[B24] Curry F. E. Michel C. C. (1980). A fiber matrix model of capillary permeability. *Microvasc. Res.* 20 96–99. 10.1016/0026-2862(80)90024-2 7412590

[B25] Curry F. R. (2005). Microvascular solute and water transport. *Microcirculation* 12 17–31. 10.1080/10739680590894993 15804971

[B26] Davies P. F. (1995). Flow-mediated endothelial mechanotransduction. *Physiol. Rev.* 75 519–560. 10.1152/physrev.1995.75.3.519 7624393 PMC3053532

[B27] de Mesy Bentley K. L. (2011). An 11-mum-thick glycocalyx?: it’s all in the technique! *Arterioscler. Thromb. Vasc. Biol.* 31 1712–1713. 10.1161/ATVBAHA.111.229849 21775768

[B28] Dimmeler S. Fleming I. Fisslthaler B. Hermann C. Busse R. Zeiher A. M. (1999). Activation of nitric oxide synthase in endothelial cells by Akt-dependent phosphorylation. *Nature* 399 601–605. 10.1038/21224 10376603

[B29] Dragovich M. A. Chester D. Fu B. M. Wu C. Xu Y. Goligorsky M. S. (2016). Mechanotransduction of the endothelial glycocalyx mediates nitric oxide production through activation of TRP channels. *Am. J. Physiol. Cell Physiol.* 311 C846–C853. 10.1152/ajpcell.00288.2015 27681180

[B30] Ebong E. E. Lopez-Quintero S. V. Rizzo V. Spray D. C. Tarbell J. M. (2014). Shear-induced endothelial NOS activation and remodeling via heparan sulfate, glypican-1, and syndecan-1. *Integr. Biol.* 6 338–347. 10.1039/C3IB40199E 24480876 PMC3996848

[B31] Ebong E. E. Macaluso F. P. Spray D. C. Tarbell J. M. (2011). Imaging the endothelial glycocalyx in vitro by rapid freezing/freeze substitution transmission electron microscopy. *Arterioscler. Thromb. Vasc. Biol.* 31 1908–1915. 10.1161/ATVBAHA.111.225268 21474821 PMC3141106

[B32] Echtermeyer F. Baciu P. C. Saoncella S. Ge Y. Goetinck P. F. (1999). Syndecan-4 core protein is sufficient for the assembly of focal adhesions and actin stress fibers. *J. Cell Sci.* 112 (Pt 20) 3433–3441. 10.1242/jcs.112.20.3433 10504292

[B33] Esko J. D. Kimata K. Lindahl U. (2009). “Proteoglycans and sulfated glycosaminoglycans,” in *Essentials of Glycobiology*, eds Nd A. Varki R. D. Cummings J. D. Esko H. H. Freeze P. (New York: Cold Spring Harbor). 20301236

[B34] Fan J. Sun Y. Xia Y. Tarbell J. M. Fu B. M. (2019). Endothelial surface glycocalyx (ESG) components and ultra-structure revealed by stochastic optical reconstruction microscopy (STORM). *Biorheology* 56 77–88. 10.3233/BIR-180204 31045510

[B35] Florian J. A. Kosky J. R. Ainslie K. Pang Z. Dull R. O. Tarbell J. M. (2003). Heparan sulfate proteoglycan is a mechanosensor on endothelial cells. *Circ. Res.* 93 e136–e142. 10.1161/01.RES.0000101744.47866.D5 14563712

[B36] Fu B. M. Tarbell J. M. (2013). Mechano-sensing and transduction by endothelial surface glycocalyx: composition, structure, and function. *Wiley Interdiscip. Rev. Syst. Biol. Med.* 5 381–390. 10.1002/wsbm.1211 23401243 PMC4157334

[B37] Gallay P. (2004). Syndecans and HIV-1 pathogenesis. *Microbes Infect.* 6 617–622. 10.1016/j.micinf.2004.02.004 15158197

[B38] Gao Y. Galis Z. S. (2021). Exploring the role of endothelial cell resilience in cardiovascular health and disease. *Arterioscler. Thromb. Vasc. Biol.* 41 179–185. 10.1161/ATVBAHA.120.314346 33086867

[B39] Glasner D. R. Ratnasiri K. Puerta-Guardo H. Espinosa D. A. Beatty P. R. Harris E. (2017). Dengue virus NS1 cytokine-independent vascular leak is dependent on endothelial glycocalyx components. *PLoS Pathog.* 13:e1006673. 10.1371/journal.ppat.1006673 29121099 PMC5679539

[B40] Gouverneur M. Spaan J. A. Pannekoek H. Fontijn R. D. Vink H. (2006). Fluid shear stress stimulates incorporation of hyaluronan into endothelial cell glycocalyx. *Am. J. Physiol. Heart Circ. Physiol.* 290 H458–H452. 10.1152/ajpheart.00592.2005 16126814

[B41] Greenwald R. A. Moy W. W. (1980). Effect of oxygen-derived free radicals on hyaluronic acid. *Arthritis Rheum.* 23 455–463. 10.1002/art.1780230408 6245661

[B42] Halliwell B. (1978). Superoxide-dependent formation of hydroxyl radicals in the presence of iron salts. its role in degradation of hyaluronic acid by a superoxide-generating system. *FEBS Lett.* 96 238–242. 10.1016/0014-5793(78)80409-8 215454

[B43] Han S. Lee S. J. Kim K. E. Lee H. S. Oh N. Park I. (2016). Amelioration of sepsis by TIE2 activation-induced vascular protection. *Sci. Transl. Med.* 8:335ra55. 10.1126/scitranslmed.aad9260 27099174

[B44] Haywood-Watson R. J. Holcomb J. B. Gonzalez E. A. Peng Z. Pati S. Park P. W. (2011). Modulation of syndecan-1 shedding after hemorrhagic shock and resuscitation. *PLoS One* 6:e23530. 10.1371/journal.pone.0023530 21886795 PMC3158765

[B45] Henry C. B. Duling B. R. (1999). Permeation of the luminal capillary glycocalyx is determined by hyaluronan. *Am. J. Physiol.* 277 H508–H514. 10.1152/ajpheart.1999.277.2.H508 10444475

[B46] Hippensteel J. A. Anderson B. J. Orfila J. E. Mcmurtry S. A. Dietz R. M. Su G. (2019a). Circulating heparan sulfate fragments mediate septic cognitive dysfunction. *J. Clin. Invest.* 129 1779–1784. 10.1172/JCI124485 30720464 PMC6436867

[B47] Hippensteel J. A. Uchimido R. Tyler P. D. Burke R. C. Han X. Zhang F. (2019b). Intravenous fluid resuscitation is associated with septic endothelial glycocalyx degradation. *Crit. Care* 23:259. 10.1186/s13054-019-2534-2 31337421 PMC6652002

[B48] Huxley V. H. Curry F. E. (1985). Albumin modulation of capillary permeability: test of an adsorption mechanism. *Am. J. Physiol.* 248 H264–H273. 10.1152/ajpheart.1985.248.2.H264 3871592

[B49] Ihrcke N. S. Wrenshall L. E. Lindman B. J. Platt J. L. (1993). Role of heparan sulfate in immune system-blood vessel interactions. *Immunol. Today* 14 500–505. 10.1016/0167-5699(93)90265-M 8274190

[B50] Jacob M. Bruegger D. Rehm M. Welsch U. Conzen P. Becker B. F. (2006). Contrasting effects of colloid and crystalloid resuscitation fluids on cardiac vascular permeability. *Anesthesiology* 104 1223–1231. 10.1097/00000542-200606000-00018 16732094

[B51] Jambusaria A. Hong Z. Zhang L. Srivastava S. Jana A. Toth P. T. (2020). Endothelial heterogeneity across distinct vascular beds during homeostasis and inflammation. *Elife* 9:e51413. 10.7554/eLife.51413 31944177 PMC7002042

[B52] Johansson P. I. Sorensen A. M. Perner A. Welling K. L. Wanscher M. Larsen C. F. (2011a). Disseminated intravascular coagulation or acute coagulopathy of trauma shock early after trauma? an observational study. *Crit. Care* 15:R272. 10.1186/cc10553 22087841 PMC3388658

[B53] Johansson P. I. Stensballe J. Rasmussen L. S. Ostrowski S. R. (2011b). A high admission syndecan-1 level, a marker of endothelial glycocalyx degradation, is associated with inflammation, protein C depletion, fibrinolysis, and increased mortality in trauma patients. *Ann. Surg.* 254 194–200. 10.1097/SLA.0b013e318226113d 21772125

[B54] Johnson G. B. Brunn G. J. Kodaira Y. Platt J. L. (2002). Receptor-mediated monitoring of tissue well-being via detection of soluble heparan sulfate by Toll-like receptor 4. *J. Immunol.* 168 5233–5239. 10.4049/jimmunol.168.10.5233 11994480

[B55] Jung F. Kruger-Genge A. Franke R. P. Hufert F. Kupper J. H. (2020). COVID-19 and the endothelium. *Clin. Hemorheol. Microcirc.* 75 7–11. 10.3233/CH-209007 32568187 PMC7458498

[B56] Kaur S. Tripathi D. M. Yadav A. (2020). The enigma of endothelium in COVID-19. *Front. Physiol.* 11:989. 10.3389/fphys.2020.00989 32848893 PMC7417426

[B57] Kozar R. A. Peng Z. Zhang R. Holcomb J. B. Pati S. Park P. (2011). Plasma restoration of endothelial glycocalyx in a rodent model of hemorrhagic shock. *Anesth. Analg.* 112 1289–1295. 10.1213/ANE.0b013e318210385c 21346161 PMC3102787

[B58] Kutuzov N. Flyvbjerg H. Lauritzen M. (2018). Contributions of the glycocalyx, endothelium, and extravascular compartment to the blood-brain barrier. *Proc. Natl. Acad. Sci. U. S. A.* 115 E9429–E9438. 10.1073/pnas.1802155115 30217895 PMC6176561

[B59] Lennon F. E. Singleton P. A. (2011). Hyaluronan regulation of vascular integrity. *Am. J. Cardiovasc. Dis.* 1 200–213. 22254199 PMC3253523

[B60] Li L. Ly M. Linhardt R. J. (2012). Proteoglycan sequence. *Mol. Biosyst.* 8 1613–1625. 10.1039/c2mb25021g 22513887 PMC3425375

[B61] Li Q. Xie Y. Wong M. Lebrilla C. B. (2019). Characterization of cell Glycocalyx with mass spectrometry methods. *Cells* 8:882. 10.3390/cells8080882 31412618 PMC6721671

[B62] Li Q. Xie Y. Wong M. Barboza M. Lebrilla C. B. (2020). Comprehensive structural glycomic characterization of the glycocalyxes of cells and tissues. *Nat. Protoc.* 15 2668–2704. 10.1038/s41596-020-0350-4 32681150 PMC11790333

[B63] Libby P. Luscher T. (2020). COVID-19 is, in the end, an endothelial disease. *Eur. Heart J.* 41 3038–3044. 10.1093/eurheartj/ehaa623 32882706 PMC7470753

[B64] Lieleg O. Baumgartel R. M. Bausch A. R. (2009). Selective filtering of particles by the extracellular matrix: an electrostatic bandpass. *Biophys. J.* 97 1569–1577. 10.1016/j.bpj.2009.07.009 19751661 PMC2749787

[B65] Lipowsky H. H. Lescanic A. (2013). The effect of doxycycline on shedding of the glycocalyx due to reactive oxygen species. *Microvasc. Res.* 90 80–85. 10.1016/j.mvr.2013.07.004 23899417 PMC3852187

[B66] Lipowsky H. H. Kovalcheck S. Zweifach B. W. (1978). The distribution of blood rheological parameters in the microvasculature of cat mesentery. *Circ. Res.* 43 738–749. 10.1161/01.RES.43.5.738 709740

[B67] Lipowsky H. H. Sah R. Lescanic A. (2011). Relative roles of doxycycline and cation chelation in endothelial glycan shedding and adhesion of leukocytes. *Am. J. Physiol. Heart Circ. Physiol.* 300 H415–H422. 10.1152/ajpheart.00923.2010 21148759 PMC3044056

[B68] Lipowsky H. H. Usami S. Chien S. (1980). In vivo measurements of “apparent viscosity” and microvessel hematocrit in the mesentery of the cat. *Microvasc. Res.* 19 297–319. 10.1016/0026-2862(80)90050-3 7382851

[B69] Lopez-Quintero S. V. Amaya R. Pahakis M. Tarbell J. M. (2009). The endothelial glycocalyx mediates shear-induced changes in hydraulic conductivity. *Am. J. Physiol. Heart Circ. Physiol.* 296 H1451–H1456. 10.1152/ajpheart.00894.2008 19286951 PMC2685345

[B70] Luft J. H. (1966). Fine structures of capillary and endocapillary layer as revealed by ruthenium red. *Fed. Proc.* 25 1773–1783. 5927412

[B71] Luker J. N. Vigiola Cruz M. Carney B. C. Day A. Moffatt L. T. Johnson L. S. (2018). Shedding of the endothelial glycocalyx is quantitatively proportional to burn injury severity. *Ann. Burns Fire Disasters* 31 17–22. 30174566 PMC6116655

[B72] Luplertlop N. Misse D. (2008). MMP cellular responses to dengue virus infection-induced vascular leakage. *Jpn. J. Infect. Dis.* 61 298–301. 18653973

[B73] Manon-Jensen T. Multhaupt H. A. Couchman J. R. (2013). Mapping of matrix metalloproteinase cleavage sites on syndecan-1 and syndecan-4 ectodomains. *FEBS J.* 280 2320–2331. 10.1111/febs.12174 23384311

[B74] Marsac D. Garcia S. Fournet A. Aguirre A. Pino K. Ferres M. (2011). Infection of human monocyte-derived dendritic cells by ANDES Hantavirus enhances pro-inflammatory state, the secretion of active MMP-9 and indirectly enhances endothelial permeability. *Virol. J.* 8:223. 10.1186/1743-422X-8-223 21569520 PMC3104372

[B75] Martinac B. (2004). Mechanosensitive ion channels: molecules of mechanotransduction. *J. Cell Sci.* 117 2449–2460. 10.1242/jcs.01232 15159450

[B76] Matrosovich M. N. Gambaryan A. S. Tuzikov A. B. Byramova N. E. Mochalova L. V. Golbraikh A. A. (1993). Probing of the receptor-binding sites of the H1 and H3 influenza A and influenza B virus hemagglutinins by synthetic and natural sialosides. *Virology* 196 111–121. 10.1006/viro.1993.1459 8356788

[B77] Mederos y Schnitzler M. Storch U. Meibers S. Nurwakagari P. Breit A. Essin K. (2008). Gq-coupled receptors as mechanosensors mediating myogenic vasoconstriction. *EMBO J.* 27 3092–3103. 10.1038/emboj.2008.233 18987636 PMC2599876

[B78] Megens R. T. Reitsma S. Schiffers P. H. Hilgers R. H. De Mey J. G. Slaaf D. W. (2007). Two-photon microscopy of vital murine elastic and muscular arteries. combined structural and functional imaging with subcellular resolution. *J. Vasc. Res.* 44 87–98. 10.1159/000098259 17192719

[B79] Mica L. Simmen H. Werner C. M. Plecko M. Keller C. Wirth S. H. (2016). Fresh frozen plasma is permissive for systemic inflammatory response syndrome, infection, and sepsis in multiple-injured patients. *Am. J. Emerg. Med.* 34 1480–1485. 10.1016/j.ajem.2016.04.041 27260556

[B80] Mochizuki S. Vink H. Hiramatsu O. Kajita T. Shigeto F. Spaan J. A. (2003). Role of hyaluronic acid glycosaminoglycans in shear-induced endothelium-derived nitric oxide release. *Am. J. Physiol. Heart Circ. Physiol.* 285 H722–H726. 10.1152/ajpheart.00691.2002 12730059

[B81] Moseley R. Waddington R. J. Embery G. (1997). Degradation of glycosaminoglycans by reactive oxygen species derived from stimulated polymorphonuclear leukocytes. *Biochim. Biophys. Acta* 1362 221–231. 10.1016/S0925-4439(97)00083-5 9540853

[B82] Moseley R. Waddington R. Evans P. Halliwell B. Embery G. (1995). The chemical modification of glycosaminoglycan structure by oxygen-derived species in vitro. *Biochim. Biophys. Acta* 1244 245–252. 10.1016/0304-4165(95)00010-9 7599140

[B83] Multhaupt H. A. Yoneda A. Whiteford J. R. Oh E. S. Lee W. Couchman J. R. (2009). Syndecan signaling: when, where and why? *J. Physiol. Pharmacol.* 60 31–38. 20083849

[B84] Nelson A. Berkestedt I. Schmidtchen A. Ljunggren L. Bodelsson M. (2008). Increased levels of glycosaminoglycans during septic shock: relation to mortality and the antibacterial actions of plasma. *Shock* 30 623–627. 10.1097/SHK.0b013e3181777da3 18497712

[B85] Nikolian V. C. Dekker S. E. Bambakidis T. Higgins G. A. Dennahy I. S. Georgoff P. E. (2018). Improvement of blood-brain barrier integrity in traumatic brain injury and hemorrhagic shock following treatment with valproic acid and fresh Frozen Plasma. *Crit. Care Med.* 46 e59–e66. 10.1097/CCM.0000000000002800 29095204

[B86] Okada H. Takemura G. Suzuki K. Oda K. Takada C. Hotta Y. (2017). Three-dimensional ultrastructure of capillary endothelial glycocalyx under normal and experimental endotoxemic conditions. *Crit. Care* 21:261. 10.1186/s13054-017-1841-8 29058634 PMC5651619

[B87] Peng Z. Pati S. Potter D. Brown R. Holcomb J. B. Grill R. (2013). Fresh frozen plasma lessens pulmonary endothelial inflammation and hyperpermeability after hemorrhagic shock and is associated with loss of syndecan 1. *Shock* 40 195–202. 10.1097/SHK.0b013e31829f91fc 23807246 PMC3764452

[B88] Pohl U. Herlan K. Huang A. Bassenge E. (1991). EDRF-mediated shear-induced dilation opposes myogenic vasoconstriction in small rabbit arteries. *Am. J. Physiol.* 261 H2016–H2023. 10.1152/ajpheart.1991.261.6.H2016 1721502

[B89] Potter D. R. Damiano E. R. (2008). The hydrodynamically relevant endothelial cell glycocalyx observed in vivo is absent in vitro. *Circ. Res.* 102 770–776. 10.1161/CIRCRESAHA.107.160226 18258858

[B90] Potter D. R. Jiang J. Damiano E. R. (2009). The recovery time course of the endothelial cell glycocalyx in vivo and its implications in vitro. *Circ. Res.* 104 1318–1325. 10.1161/CIRCRESAHA.108.191585 19443840 PMC2764238

[B91] Pries A. R. Kuebler W. M. (2006). Normal endothelium. in: moncasa, S., higgs, A. the vascular endothelium I. *Handb. Exp. Pharmacol.* 176 1–40. 10.1007/3-540-32967-6_1 16999215

[B92] Puerta-Guardo H. Glasner D. R. Harris E. (2016). Dengue virus NS1 disrupts the endothelial glycocalyx, leading to hyperpermeability. *PLoS Pathog.* 12:e1005738. 10.1371/journal.ppat.1005738 27416066 PMC4944995

[B93] Rehm M. Zahler S. Lotsch M. Welsch U. Conzen P. Jacob M. (2004). Endothelial glycocalyx as an additional barrier determining extravasation of 6% hydroxyethyl starch or 5% albumin solutions in the coronary vascular bed. *Anesthesiology* 100 1211–1223. 10.1097/00000542-200405000-00025 15114220

[B94] Reitsma S. Slaaf D. W. Vink H. Van Zandvoort M. A. Oude Egbrink M. G. (2007). The endothelial glycocalyx: composition, functions, and visualization. *Pflugers Arch.* 454 345–359. 10.1007/s00424-007-0212-8 17256154 PMC1915585

[B95] Riley N. M. Bertozzi C. R. Pitteri S. J. (2020). A pragmatic guide to enrichment strategies for mass spectrometry-based glycoproteomics. *Mol. Cell. Proteomics* 20:100029. 10.1074/mcp.R120.002277 33583771 PMC8724846

[B96] Ringer P. Colo G. Fassler R. Grashoff C. (2017). Sensing the mechano-chemical properties of the extracellular matrix. *Matrix Biol.* 64 6–16. 10.1016/j.matbio.2017.03.004 28389162

[B97] Rizzo V. Mcintosh D. P. Oh P. Schnitzer J. E. (1998). In situ flow activates endothelial nitric oxide synthase in luminal caveolae of endothelium with rapid caveolin dissociation and calmodulin association. *J. Biol. Chem.* 273 34724–34729. 10.1074/jbc.273.52.34724 9856995

[B98] Roth J. (1983). Application of lectin–gold complexes for electron microscopic localization of glycoconjugates on thin sections. *J. Histochem. Cytochem.* 31 987–999. 10.1177/31.8.6190857 6190857

[B99] Russell R. J. Kerry P. S. Stevens D. J. Steinhauer D. A. Martin S. R. Gamblin S. J. (2008). Structure of influenza hemagglutinin in complex with an inhibitor of membrane fusion. *Proc. Natl. Acad. Sci. U. S. A.* 105 17736–17741. 10.1073/pnas.0807142105 19004788 PMC2584702

[B100] Sallisalmi M. Tenhunen J. Yang R. Oksala N. Pettila V. (2012). Vascular adhesion protein-1 and syndecan-1 in septic shock. *Acta Anaesthesiol. Scand.* 56 316–322. 10.1111/j.1399-6576.2011.02578.x 22150439

[B101] Saphire A. C. Bobardt M. D. Zhang Z. David G. Gallay P. A. (2001). Syndecans serve as attachment receptors for human immunodeficiency virus type 1 on macrophages. *J. Virol.* 75 9187–9200. 10.1128/JVI.75.19.9187-9200.2001 11533182 PMC114487

[B102] Schmidt E. P. Yang Y. Janssen W. J. Gandjeva A. Perez M. J. Barthel L. (2012). The pulmonary endothelial glycocalyx regulates neutrophil adhesion and lung injury during experimental sepsis. *Nat. Med.* 18 1217–1223. 10.1038/nm.2843 22820644 PMC3723751

[B103] Schnitzer J. E. (1988). Glycocalyx electrostatic potential profile analysis: ion, pH, steric, and charge effects. *Yale J. Biol. Med.* 61 427–446. 2462311 PMC2590420

[B104] Singh A. Ramnath R. D. Foster R. R. Wylie E. C. Friden V. Dasgupta I. (2013). Reactive oxygen species modulate the barrier function of the human glomerular endothelial glycocalyx. *PLoS One* 8:e55852. 10.1371/journal.pone.0055852 23457483 PMC3573029

[B105] Stringer S. E. Gallagher J. T. (1997). Heparan sulphate. *Int. J. Biochem. Cell Biol.* 29 709–714. 10.1016/S1357-2725(96)00170-7 9251237

[B106] Suwarto S. Sasmono R. T. Sinto R. Ibrahim E. Suryamin M. (2017). Association of endothelial glycocalyx and tight and adherens junctions with severity of plasma leakage in dengue infection. *J. Infect. Dis.* 215 992–999. 10.1093/infdis/jix041 28453844 PMC5407050

[B107] Suzuki Y. (2003). [Receptor binding specificity of influenza virus and its budding from the host cells]. *Tanpakushitsu Kakusan Koso* 48 1141–1146. 12807021

[B108] Tang T. H. Alonso S. Ng L. F. Thein T. L. Pang V. J. Leo Y. S. (2017). Increased serum hyaluronic acid and heparan sulfate in dengue fever: association with plasma leakage and disease severity. *Sci. Rep.* 7:46191. 10.1038/srep46191 28393899 PMC5385535

[B109] Tarbell J. M. Ebong E. E. (2008). The endothelial glycocalyx: a mechano-sensor and -transducer. *Sci. Signal.* 1:t8. 10.1126/scisignal.140pt8 18840877

[B110] Tarbell J. M. Pahakis M. Y. (2006). Mechanotransduction and the glycocalyx. *J. Intern. Med.* 259 339–350. 10.1111/j.1365-2796.2006.01620.x 16594902

[B111] Teuwen L. A. Geldhof V. Pasut A. Carmeliet P. (2020). COVID-19: the vasculature unleashed. *Nat. Rev. Immunol.* 20 389–391. 10.1038/s41577-020-0343-0 32439870 PMC7240244

[B112] Uchimido R. Schmidt E. P. Shapiro N. I. (2019). The glycocalyx: a novel diagnostic and therapeutic target in sepsis. *Crit. Care* 23:16. 10.1186/s13054-018-2292-6 30654825 PMC6337861

[B113] van den Berg B. M. Vink H. Spaan J. A. (2003). The endothelial glycocalyx protects against myocardial edema. *Circ. Res.* 92 592–594. 10.1161/01.RES.0000065917.53950.75 12637366

[B114] van Haaren P. M. Vanbavel E. Vink H. Spaan J. A. (2003). Localization of the permeability barrier to solutes in isolated arteries by confocal microscopy. *Am. J. Physiol. Heart Circ. Physiol.* 285 H2848–H2856. 10.1152/ajpheart.00117.2003 12907418

[B115] Vink H. Duling B. R. (2000). Capillary endothelial surface layer selectively reduces plasma solute distribution volume. *Am. J. Physiol. Heart Circ. Physiol.* 278 H285–H289. 10.1152/ajpheart.2000.278.1.H285 10644610

[B116] Vink H. Constantinescu A. A. Spaan J. A. (2000). Oxidized lipoproteins degrade the endothelial surface layer : implications for platelet-endothelial cell adhesion. *Circulation* 101 1500–1502. 10.1161/01.CIR.101.13.1500 10747340

[B117] Voyvodic P. L. Min D. Liu R. Williams E. Chitalia V. Dunn A. K. (2014). Loss of syndecan-1 induces a pro-inflammatory phenotype in endothelial cells with a dysregulated response to atheroprotective flow. *J. Biol. Chem.* 289 9547–9559. 10.1074/jbc.M113.541573 24554698 PMC3975006

[B118] Wang C. Puerta-Guardo H. Biering S. B. Glasner D. R. Tran E. B. Patana M. (2019). Endocytosis of flavivirus NS1 is required for NS1-mediated endothelial hyperpermeability and is abolished by a single N-glycosylation site mutation. *PLoS Pathog.* 15:e1007938. 10.1371/journal.ppat.1007938 31356638 PMC6687192

[B119] Weinbaum S. Tarbell J. M. Damiano E. R. (2007). The structure and function of the endothelial glycocalyx layer. *Annu. Rev. Biomed. Eng.* 9 121–167. 10.1146/annurev.bioeng.9.060906.151959 17373886

[B120] Weis W. Brown J. H. Cusack S. Paulson J. C. Skehel J. J. Wiley D. C. (1988). Structure of the influenza virus haemagglutinin complexed with its receptor, sialic acid. *Nature* 333 426–431. 10.1038/333426a0 3374584

[B121] Xu D. Young J. H. Krahn J. M. Song D. Corbett K. D. Chazin W. J. (2013). Stable RAGE-heparan sulfate complexes are essential for signal transduction. *ACS Chem. Biol.* 8 1611–1620. 10.1021/cb4001553 23679870 PMC3806902

[B122] Xu D. Young J. Song D. Esko J. D. (2011). Heparan sulfate is essential for high mobility group protein 1 (HMGB1) signaling by the receptor for advanced glycation end products (RAGE). *J. Biol. Chem.* 286 41736–41744. 10.1074/jbc.M111.299685 21990362 PMC3308882

[B123] Yamaoka-Tojo M. (2020). Endothelial glycocalyx damage as a systemic inflammatory microvascular endotheliopathy in COVID-19. *Biomed. J.* 43 399–413. 10.1016/j.bj.2020.08.007 33032965 PMC7443638

[B124] Yang X. Meegan J. E. Jannaway M. Coleman D. C. Yuan S. Y. (2018). A disintegrin and metalloproteinase 15-mediated glycocalyx shedding contributes to vascular leakage during inflammation. *Cardiovasc. Res.* 114 1752–1763. 10.1093/cvr/cvy167 29939250 PMC6198742

[B125] Yen W. Cai B. Yang J. Zhang L. Zeng M. Tarbell J. M. (2015). Endothelial surface glycocalyx can regulate flow-induced nitric oxide production in microvessels in vivo. *PLoS One* 10:e0117133. 10.1371/journal.pone.0117133 25575016 PMC4289188

[B126] Yoon J. H. Lee E. S. Jeong Y. (2017). In vivo imaging of the cerebral endothelial Glycocalyx in mice. *J. Vasc. Res.* 54 59–67. 10.1159/000457799 28365703

[B127] Zehtabchi S. Nishijima D. K. (2009). Impact of transfusion of fresh-frozen plasma and packed red blood cells in a 1:1 ratio on survival of emergency department patients with severe trauma. *Acad. Emerg. Med.* 16 371–378. 10.1111/j.1553-2712.2009.00386.x 19302364

[B128] Zeng Y. Liu J. (2016). Role of glypican-1 in endothelial NOS activation under various steady shear stress magnitudes. *Exp. Cell Res.* 348 184–189. 10.1016/j.yexcr.2016.09.017 27688027

[B129] Zeng Y. Tarbell J. M. (2014). The adaptive remodeling of endothelial glycocalyx in response to fluid shear stress. *PLoS One* 9:e86249. 10.1371/journal.pone.0086249 24465988 PMC3896483

[B130] Zeng Y. Zhang X. F. Fu B. M. Tarbell J. M. (2018). The role of endothelial surface Glycocalyx in mechanosensing and transduction. *Adv. Exp. Med. Biol.* 1097 1–27. 10.1007/978-3-319-96445-4_1 30315537

[B131] Zou Y. Akazawa H. Qin Y. Sano M. Takano H. Minamino T. (2004). Mechanical stress activates angiotensin II type 1 receptor without the involvement of angiotensin II. *Nat. Cell Biol.* 6 499–506. 10.1038/ncb1137 15146194

